# Equivalence of laboratory-developed test and PD-L1 IHC 22C3 pharmDx across all combined positive score indications

**DOI:** 10.1371/journal.pone.0285764

**Published:** 2023-06-02

**Authors:** Gilad Vainer, Lingkang Huang, Kenneth Emancipator, Shanthy Nuti

**Affiliations:** 1 Department of Pathology, Hadassah Medical Center, Hebrew University of Jerusalem, Jerusalem, Israel; 2 Department of Biostatistics and Research Decision Sciences, Merck & Co., Inc., Rahway, New Jersey, United States of America; 3 Department of Translational Medicine, Merck & Co., Inc., Rahway, New Jersey, United States of America; 4 Biomarkers and Diagnostics, Oncology, Global Medical and Scientific Affairs, MRL, Merck & Co., Inc., Rahway, New Jersey, United States of America; Postgraduate Institute of Medical Education and Research, INDIA

## Abstract

We conducted an analysis across multiple PD-L1 combined positive score (CPS) indications to establish concordance of a 22C3 antibody–based laboratory-developed test (LDT) on the Ventana BenchMark XT or BenchMark ULTRA platform and the regulatory-approved PD-L1 IHC 22C3 pharmDx in cervical cancer (CC), esophageal squamous cell carcinoma (ESCC), head and neck squamous cell carcinoma (HNSCC), triple-negative breast cancer (TNBC), and urothelial carcinoma (UC). Tumor specimens from each tumor type were stained with 22C3 antibody and scored using the 22C3 antibody–based LDT, and scores were compared with those using PD-L1 IHC 22C3 pharmDx. PD-L1 status was measured by the pathologist using CPS as a continuous score and using clinically relevant cutoffs (CC, ≥1 and ≥10; HNSCC, ≥1 and ≥20; ESCC, TNBC, and UC, ≥10). The agreement between the BenchMark platforms and PD-L1 IHC 22C3 pharmDx was assessed by intraclass correlation coefficient (ICC) and a contingency table for clinical interpretation. A total of 522 samples were evaluated for the pan-tumor analysis (CC, n = 77; ESCC, n = 80; HNSCC, n = 126; TNBC, n = 118, UC, n = 121). Most clinical interpretations of PD-L1 status were concordant between the BenchMark XT and PD-L1 IHC 22C3 pharmDx for all five tumor types with regard to negative percentage agreement (NPA; 83–97%), positive percentage agreement (PPA; 86–100%), and overall percentage agreement (OPA; 90–97%); the ICC by tumor type was high (≥0.88). Importantly, the pan-tumor ICC was 0.95 (95% CI 0.94–0.96). Thirty additional TNBC samples were evaluated using the BenchMark ULTRA and PD-L1 IHC 22C3 pharmDx; the NPA, PPA, and OPA were 100%. The 22C3 antibody–based LDT on Ventana BenchMark XT and BenchMark ULTRA platforms demonstrated high concordance with the regulatory-approved PD-L1 IHC 22C3 pharmDx across multiple tumor types. These findings suggest the comparability of PD-L1 IHC 22C3 pharmDx with an LDT based on the 22C3 antibody.

## Introduction

Pembrolizumab is a highly selective humanized monoclonal antibody that blocks the interaction between programmed death 1 (PD-1) and its ligands, PD-L1 and PD-L2, which helps restore T-cell responses against tumor cells [1–4]. The antitumor activity and safety of pembrolizumab have been established across a spectrum of solid and hematologic malignancies, which has led to its approval by the US Food and Drug Administration (FDA) and European Medicines Agency for these types of cancer [3, 4]. PD-L1 expression is predictive of response to PD-1/PD-L1 inhibitors in several tumor types [5–7]. Accordingly, several indications for which pembrolizumab is approved by regulatory agencies are specifically for patients whose tumors express PD-L1 above certain thresholds as determined by an FDA-approved and/or CE-marked companion diagnostic, PD-L1 IHC 22C3 pharmDx (Agilent, Carpinteria, CA) [3, 4, 8]. For example, pembrolizumab is approved for the treatment of patients with recurrent or metastatic cervical cancer with disease progression on or after chemotherapy whose tumors express PD-L1 combined positive score (CPS) ≥1, as determined by PD-L1 IHC 22C3 pharmDx [3, 8]. PD-L1 IHC 22C3 pharmDx is also clinically validated and approved by the FDA in selecting patients for pembrolizumab monotherapy or combination therapies who have non–small cell lung cancer (NSCLC; determined using tumor proportion score [TPS]), head and neck squamous cell carcinoma (HNSCC), esophageal squamous cell carcinoma (ESCC), and triple-negative breast cancer (TNBC) [3, 8]. Similarly, several indications for pembrolizumab approved by the European Medicines Agency are specifically for patients whose tumors express PD-L1 by a validated test [4].

PD-L1 IHC 22C3 pharmDx is a qualitative immunohistochemical (IHC) assay using the monoclonal anti–PD-L1 clone 22C3 to detect PD-L1 protein in formalin-fixed, paraffin-embedded (FFPE) tumor tissues and is designed for use on the Dako Autostainer Link 48 (Agilent) [8]. However, many pathology laboratories do not have access to the Autostainer Link 48 and therefore cannot use PD-L1 IHC 22C3 pharmDx to assess the PD-L1 tumor status of patients who may be candidates for treatment with pembrolizumab. These laboratories often assess PD-L1 status using the 22C3 monoclonal antibody–based tests and laboratory-developed tests (LDTs) on the Ventana BenchMark XT or Benchmark ULTRA (Roche Diagnostics, Basel, Switzerland), both widely available IHC platforms. However, data on the performance of these LDTs compared with PD-L1 IHC 22C3 pharmDx are scant.

An analytical harmonization study, which fits the 22C3 monoclonal antibody to the Ventana BenchMark XT platform, has been published [9]. High concordance between PD-L1 assessments in NSCLC samples (scored by TPS measuring PD-L1 in tumor cells only) and assessments made using PD-L1 IHC 22C3 pharmDx was demonstrated using an LDT [9]; the scoring algorithm with CPS (PD-L1 in tumor cells and tumor-associated lymphocytes and macrophages) was not evaluated.

The aim of the current study was to establish the concordance between the 22C3 antibody–based LDT and the gold standard PD-L1 IHC 22C3 pharmDx using CPS in five tumor types separately (cervical cancer [CC], ESCC, HNSCC, TNBC, and urothelial carcinoma [UC]) and together in a pan-tumor analysis.

## Materials and methods

### Tumor samples

Archival FFPE tumor blocks from patients with CC, ESCC, HNSCC, TNBC, and UC were sourced from various suppliers Precision for Medicine, BioIVT, and MTgroup). Samples considered eligible for this study were <7 years old per technical specifications and had preserved tumor morphology, at least 1.5 × 1.5 cm of tumor, necrosis <20%, tumor cellularity >50%, and sufficient thickness for three separate cutting sessions. IHC testing was performed between 2019 and 2021 at the Hadassah Hebrew University Medical Center in Jerusalem, Israel. This analytical study used fully anonymized human tissue samples sourced from various suppliers; patient informed consent and approval by an institutional review board or an independent ethics committee were not required.

### Immunohistochemistry

Archival FFPE tissue blocks were sectioned at a 4-μm thickness and attached to positively charged glass slides; slides were stored at room temperature. Staining was performed using consecutive serial sections ≤14 days after sectioning. PD-L1 IHC 22C3 pharmDx was performed on the Autostainer Link 48 platform following the manufacturer’s specifications [8].

For the LDT, samples were stained with the 22C3 antibody (Agilent) diluted 1:33 in antibody diluent with casein using the BenchMark XT platform (Ventana). The LDT used for this analysis has been previously described [9]. Characteristics of the protocol using the 22C3 antibody on the BenchMark XT platform are summarized in Table 1. Briefly, the protocol used the ultraView kit (with amplification), and the following steps were performed: (1) specimens prepared in FFPE glass slides were selected (the “on” option on the machine was checked), (2) specimens were deparaffinized, (3) cell condition 1 was selected for 60 minutes, (4) primary antibody was incubated at 37°C for 1 hour, (5) ultraView amplification was selected, and (6) specimens were counterstained with one drop of hematoxylin II for 4 minutes to provide very light nuclear details. Characteristics of the protocol using the 22C3 antibody on the BenchMark ULTRA platform were the same, with the exception of step 5, which included selecting amplification as well as mouse antibody amplification owing to the newer technology. This protocol was rigorously optimized and clinically tested as previously described [9]. The intensity and specificity were calibrated using the Agilent analytic controls and normal human tonsil tissue. Human tonsil tissue served as a benchmark and as on-slide controls for each clinical case.

**Table 1:** Characteristics of the BenchMark XT platform protocol.

Characteristic	Description
Specimen thickness/ preparation	FFPE 4 μm/positively charged glass slide^a^ Use tonsil tissue as positive and dynamic-range control on every slide^b^
Instrument/platform detection	BenchMark XT, ultraView V3 kit with amplification
Antibody	Concentrated 22C3
Dilution	1:33 to 1:50^c^
Cell conditioning	Cell condition 1 standard
Antibody incubation	37 °C; 1 hour
Amplification	Selected
Counterstain	Hemotoxylin II, 4 minutes^d^

Note: FFPE, formalin-fixed paraffin-embedded; PD-L1, programmed death ligand 1.

^a^It is best to use freshly made cuts (up to 2 weeks old), stored at room temperature.

^b^Tonsil tissue should be used to judge the dynamic range of the PD-L1 staining; briefly, the macrophages of the germinal centers should be lightly stained at ×4 magnification and readily visible at ×10 magnification.

^c^Best freshly made with manual titration with Dako antibody diluent or Ventana Discovery diluent (stored up to 1 week at 4 °C).

^d^Light, delicate hematoxylin is important.

### Analysis

To minimize interobserver bias, all samples were reviewed, analyzed, and scored for PD-L1 CPS (score range, 0–100) by one trained pathologist (GWV) according to the same CPS clinical algorithm for PD-L1 IHC 22C3 pharmDx. First, the slides (per each indication) were stained using the Autostainer Link 48 platform and evaluated. Each slide was evaluated twice, with a washout period of 1 week. The final score was the average of both readings. Then, after a mandatory washout period of ≥3 weeks, the process was repeated for the LDT-stained slides, as before. The final BenchMark XT or BenchMark ULTRA platform–based LDT score was the average of both readings. Only after the reading was complete was the final score that was obtained using the LDT compared with that obtained using the gold standard (PD-L1 IHC 22C3 pharmDx).

Scatterplots of CPS as a continuous variable were generated using GraphPad Prism 6 (GraphPad Software, La Jolla, CA). The intraclass correlation coefficient (ICC) and Spearman’s correlation coefficient (*ρ*) were used to analyze the concordance of CPS by the 22C3 antibody–based LDT on the BenchMark XT platform versus PD-L1 IHC 22C3 pharmDx. Analyses were performed by each individual tumor type and in a pan-tumor analysis. For an additional 30 TNBC samples, ICC and Spearman’s *ρ* were used to analyze the concordance of CPS by the 22C3 antibody–based LDT on the BenchMark ULTRA platform versus PD-L1 IHC 22C3 pharmDx.

To determine PD-L1 status in this study, each CPS was transformed to clinical groups using the following standard clinical CPS cutoffs indicated with PD-L1 IHC 22C3 pharmDx: CPS <1 (negative), CPS ≥1 (positive), and CPS ≥10 (positive) for CC samples; CPS <10 (negative) and CPS ≥10 (positive) for ESCC, TNBC, and UC samples; CPS <1 (negative), CPS ≥1 (positive), and CPS ≥20 (positive) for HNSCC samples [8, 10]. After PD-L1 status was determined using the LDT and PD-L1 IHC 22C3 pharmDx, the agreement between both assays was characterized with overall percentage agreement (OPA), positive percentage agreement (PPA), and negative percentage agreement (NPA) using a contingency table. OPA was defined as the number of agreed samples divided by the total number of samples. PPA was defined as the number of positive samples identified by both LDT and PD-L1 IHC 22C3 pharmDx divided by the total number of positive samples by PD-L1 IHC 22C3 pharmDx. Similarly, NPA was defined as the number of negative samples identified by both LDT and PD-L1 IHC 22C3 pharmDx divided by the total number of negative samples by PD-L1 IHC 22C3 pharmDx. Notably, NPA was not calculated for cervical cancer samples with CPS ≥1 because of the known and expected low number of PD-L1–negative cervical cancer samples; this sole constraint was decided before the analysis.

## Results

### Analytic harmonization

**Fig 1:**
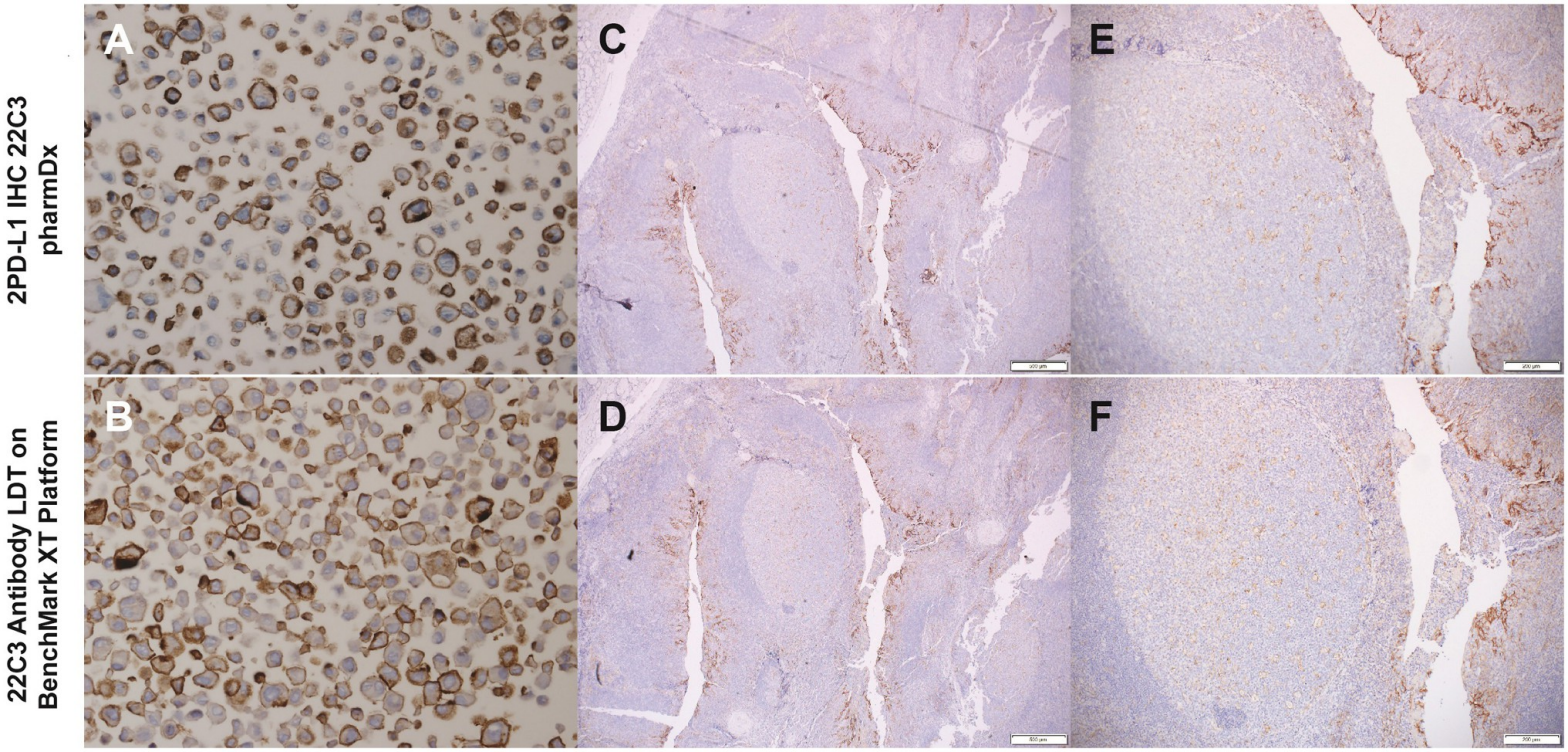
PD-L1 staining pattern using PD-L1 IHC 22C3 pharmDx and the 22C3 antibody–based LDT on the BenchMark XT platform. (A, B) Analytic PD-L1–positive slide from Agilent. (C–F) PD-L1 staining pattern in human tonsil tissue. (E, F) Dynamic range of the germinal center macrophages (light-brown colored, IHC score +1) and the intense staining of the invaginated epithelium (dark-brown colored, IHC score +3) in serial samples. LDT, laboratory-developed test; PD-L1, programmed death ligand 1.

pharmDx-positive control slides from Agilent were first used as analytic controls for both IHC platforms (Fig 1A). Because pathology laboratories without access to the Autostainer Link 48 platform cannot use this control, we also stained human tonsil tissue using PD-L1 IHC 22C3 pharmDx. Both resulted in a wide dynamic range of PD-L1 staining (Fig 1A and 1B). In the tonsil tissue samples, staining ranged from an intense dark-brown colored staining of the invaginated epithelium (indicative of strong PD-L1 expression; IHC score +3) to a light-brown colored staining of the germinal center macrophages (indicative of weaker PD-L1 expression; IHC score +1) (Fig 1C and 1D). Importantly, staining the Agilent analytic control slide with the 22C3 antibody–based LDT on the BenchMark XT platform resulted in a very similar staining pattern (Fig 1C and 1D), as did staining serial cuts of human tonsil tissue (Fig 1E and 1F). Notably, because of the wide availability of human tonsil tissue, it served as an on-slide positive control for all the slides in this study.

### Cervical cancer

**Fig 2:**
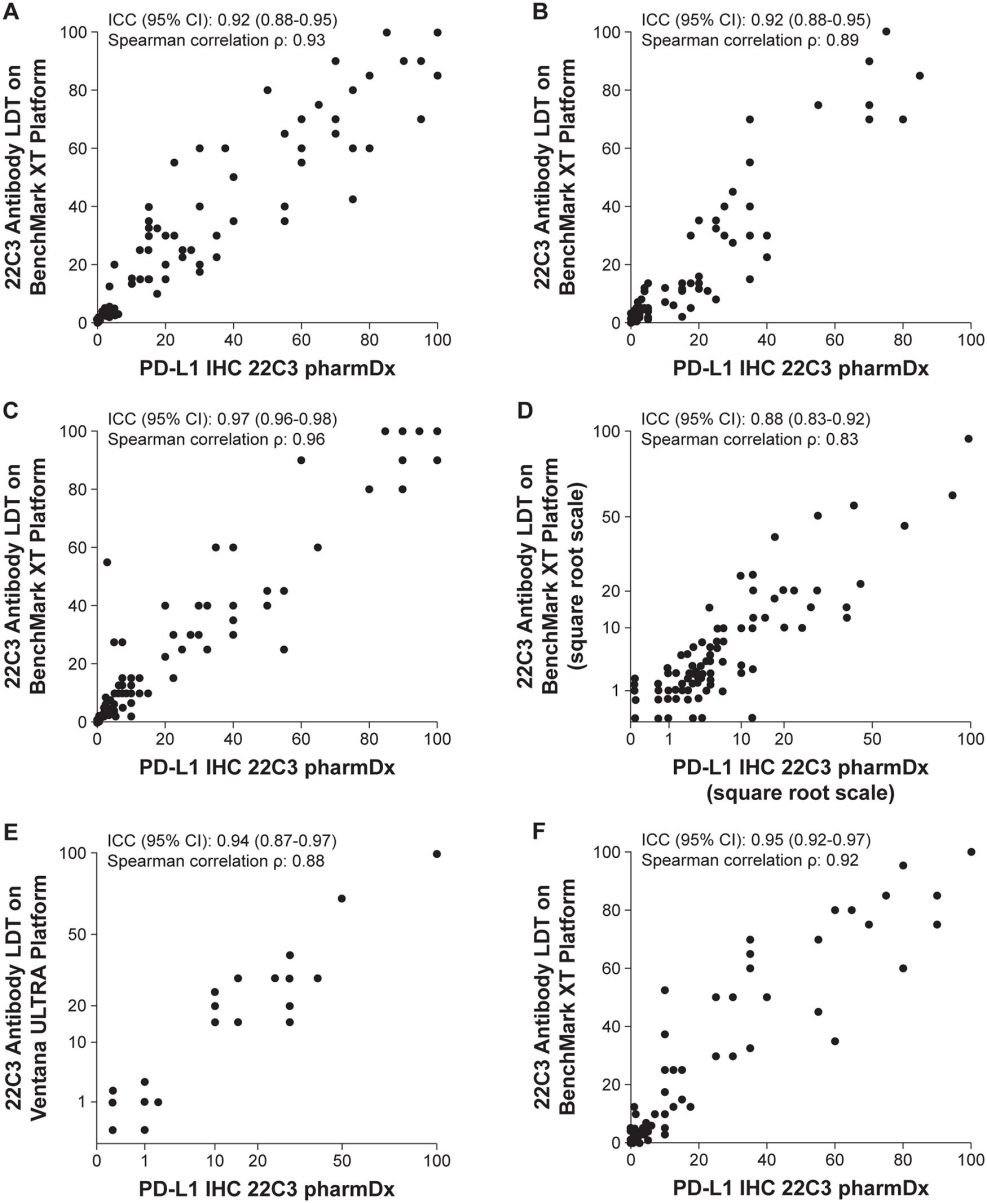
Correlation of PD-L1 CPS as a continuous variable between the 22C3 antibody–based LDT on the BenchMark XT or the BenchMark ULTRA platform and PD-L1 IHC 22C3 pharmDx for the following samples: (A) CC (n = 77), (B) ESCC (n = 80), (C) HNSCC (n = 126), (D) TNBC using the BenchMark XT platform (n = 118), (E) TNBC using the BenchMark ULTRA platform (n = 30), and (F) UC (n = 121). CC, cervical cancer; CPS, combined positive score; ESCC, esophageal squamous cell carcinoma; HNSCC, head and neck squamous cell carcinoma; ICC, intraclass correlation coefficient; IHC, immunohistochemistry; LDT, laboratory-developed test; PD-L1, programmed death ligand 1; TNBC, triple-negative breast cancer; UC, urothelial carcinoma.

A total of 77 CC tumor samples were analyzed. The correlation of PD-L1 CPS showed an ICC coefficient of 0.92 (Spearman’s *ρ* = 0.93) (Fig 2A). All CC tumor samples were in agreement for CPS ≥1 (71 of 71) and CPS ≥10 (55 of 55), with OPA rates of 96% (95% confidence interval [CI], 89–99%) and 97% (95% CI, 91–99%), respectively (Table 2).

**Table 2:** Clinical interpretation of PD-L1 status using the 22C3 antibody–based LDT on the BenchMark XT or BenchMark ULTRA platform and PD-L1 IHC 22C3 pharmDx.

Tumor type	BenchMark platform	PD-L1 CPS cutoff	NPA		PPA		OPA	
			n/N	% (95% CI)	n/N	% (95% CI)	n/N	% (95% CI)
CC	XT	≥1	—	—	71/71	100 (95–100)	74/77	96 (89–99)
		≥10	20/22	91 (72–97)	55/55	100 (93–100)	75/77	97 (91–99)
ESCC	XT	≥10	36/39	92 (80–97)	36/41	88 (74–95)	72/80	90 (81–95)
HNSCC	XT	≥1	20/24	83 (61–93)	101/102	99 (95–100)	121/126	96 (91–98)
		≥20	74/77	96 (89–99)	48/49	98 (89–100)	122/126	97 (92–99)
TNBC	XT	≥10	86/89	97 (91–99)	25/29	86 (69–95)	111/118	94 (88–97)
	ULTRA		13/13	100 (77–100)	17/17	100 (82–100)	30/30	100 (89–100)
UC	XT	≥10	77/80	96 (90–99)	39/41	95 (84–99)	116/121	96 (91–98)

Note: CC, cervical cancer; CPS, combined positive score; ESCC, esophageal squamous cell carcinoma; HNSCC, head and neck squamous cell carcinoma; LDT, laboratory-developed test; NPA, negative percentage agreement; OPA, overall percentage agreement; PD-L1, programmed death ligand 1; PPA, positive percentage agreement; TNBC, triple-negative breast cancer; UC, urothelial carcinoma.

### Esophageal squamous cell carcinoma

A total of 80 ESCC samples were analyzed. The correlation of PD-L1 CPS showed an ICC coefficient of 0.92 (Spearman’s *ρ* = 0.89) (Fig 2B). Of 41 ESCC samples, 36 were in agreement for CPS ≥10, with an OPA rate of 90% (95% CI, 81–95%) (Table 2).

### Head and neck squamous cell carcinoma

A total of 126 HNSCC samples were analyzed. The correlation of PD-L1 CPS showed an ICC coefficient of 0.97 (Spearman’s *ρ* = 0.96) (Fig 2C). Almost all HNSCC samples were in agreement for CPS ≥1 (101 of 102) and CPS ≥20 (48 of 49), resulting in OPA rates of 96% (95% CI, 91–98%) and 97% (95% CI, 92–99%), respectively (Table 2).

### Triple-negative breast cancer

A total of 118 TNBC samples were analyzed with the BenchMark XT platform. The correlation for PD-L1 CPS showed an ICC coefficient of 0.88 (Spearman’s *ρ* = 0.83) (Fig 2D). Almost all TNBC samples were in agreement for CPS ≥10 (111 of 118) for an OPA rate of 94% (95% CI, 88–97%) (Table 2). An additional 30 samples were analyzed using the BenchMark ULTRA platform. The correlation for CPS showed an ICC coefficient of 0.94 (Spearman’s *ρ* = 0.88) (Fig 2E). All samples were in agreement for CPS ≥10, for an OPA rate of 100% (Table 2).

### Urothelial carcinoma

A total of 121 UC samples were analyzed. The correlation of PD-L1 CPS showed an ICC coefficient of 0.95 (Spearman’s *ρ* = 0.92) (Fig 2F). Almost all UC samples were in agreement (39 of 41), with an OPA rate of 96% (95% CI, 91–98%) (Table 2).

### Pan-tumor analysis

**Fig 3:**
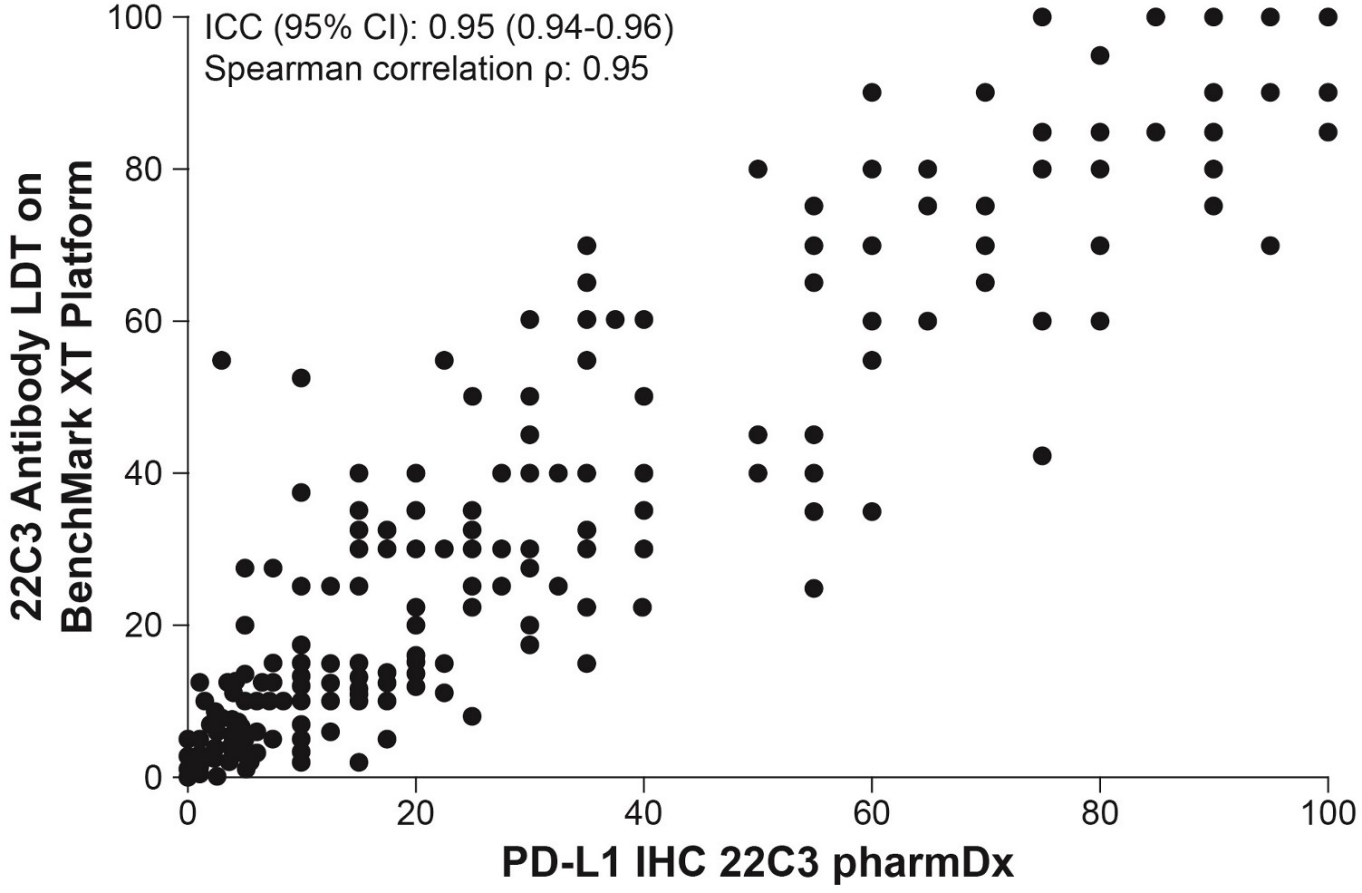
Pan-tumor (N = 522) correlation of PD-L1 CPS as a continuous variable between the 22C3 antibody–based LDT on the BenchMark XT platform and PD-L1 IHC 22C3 pharmDx. CPS, combined positive score; ICC, intraclass correlation coefficient; IHC, immunohistochemistry; LDT, laboratory-developed test; PD-L1, programmed death ligand 1.

A total of 522 samples of various tumor types were acquired and included in the pan-tumor analysis. The correlation of PD-L1 CPS showed an ICC coefficient of 0.95 (Spearman’s *ρ* = 0.93) (Fig 3).

### Qualitative comparison

**Fig 4:**
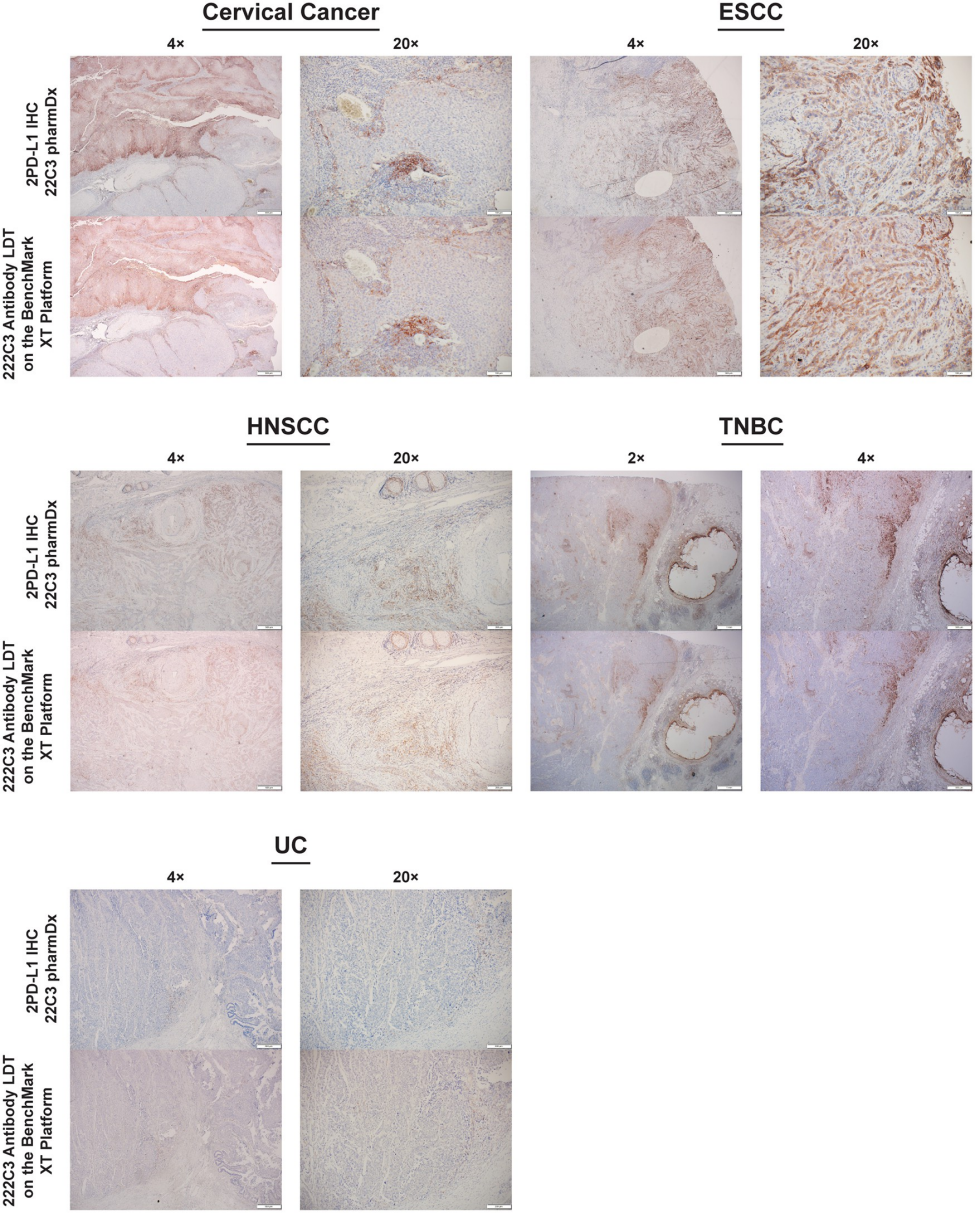
PD-L1 staining pattern using PD-L1 IHC 22C3 pharmDx and the 22C3 antibody–based LDT on the BenchMark XT platform in CC, ESCC, HNSCC, TNBC, and UC. CC, cervical cancer; ESCC, esophageal squamous cell carcinoma; HNSCC, head and neck squamous cell carcinoma; LDT, laboratory-developed test; PD-L1, programmed death ligand 1; TNBC, triple-negative breast cancer; UC, urothelial carcinoma.

As with the analytic control, morphologic evaluation of stained tissue samples showed high similarity of PD-L1 staining patterns and intensities between PD-L1 IHC 22C3 pharmDx and the 22C3 antibody–based LDT on the BenchMark XT platform in CC, ESCC, HNSCC, TNBC, and UC samples (Fig 4).

## Discussion and conclusions

Identifying patients who are most likely to benefit from a given therapy based on biomarkers is increasingly important in creating a personalized treatment plan in oncology. Although anti–PD-1/PD-L1 immunotherapy has improved the prognosis substantially across a spectrum of cancers, some patients do not respond to therapy [11]. Although a regulatory-approved companion diagnostic and a validated assay to facilitate patient selection for pembrolizumab, access to PD-L1 IHC 22C3 pharmDx is limited in some regions because of the lower presence of the assay platform, namely the Dako Autostainer Link 48, particularly outside the United States. Therefore, there is an urgent need for a validated LDT protocol that is easily adoptable using the widely available Ventana BenchMark XT or BenchMark ULTRA platforms and that produces reliable PD-L1 results.

Previous studies comparing validated PD-L1 IHC assays and PD-L1 LDTs based on different anti–PD-L1 antibodies have reported inconsistent and divergent results [12–16]. These inconsistencies may be partly attributed to several factors, including the following: small tumor sample size, choice of control tissue, potential variability in staining intensity of immune cells, variable staining intensity of immune/tumor cells for different antibody clones, and the potential subjective nature of PD-L1 scoring algorithms (i.e., clinically relevant cutoffs), which can give rise to interobserver variability in PD-L1 assessment by pathologists [12–15]. A recent meta-analysis assessing the diagnostic accuracy and interchangeability of PD-L1 IHC assays also suggested that when the testing laboratory is not able to use the regulatory-approved companion diagnostic for PD-L1 assessment for its specific purpose, use of a validated LDT developed for the same purpose as the original PD-L1 regulatory-approved companion diagnostic is better than switching to another PD-L1 regulatory-approved companion diagnostic developed for a different purpose [16].

Using both a 22C3 antibody–based LDT on the Ventana BenchMark XT platform previously validated for NSCLC and the PD-L1 CPS algorithm, we evaluated PD-L1 expression in tumor samples from patients with CC, ESCC, HNSCC, TNBC, and UC and compared it with assessments using the gold standard, the regulatory-approved PD-L1 IHC 22C3 pharmDx. In our study, CPS as a continuous variable demonstrated a high correlation between the 22C3 antibody–based LDT on the BenchMark XT platform and PD-L1 IHC 22C3 pharmDx across all assessed tumor types. A high correlation between the 22C3 antibody–based LDT on the BenchMark ULTRA platform and PD-L1 IHC 22C3 pharmDx was observed in a small set of TNBC samples. Furthermore, high concordance between the 22C3 antibody–based LDT on the BenchMark XT or BenchMark ULTRA platform and PD-L1 IHC 22C3 pharmDx was also reported for the clinical interpretations of PD-L1 status by tumor type. These findings suggest that this 22C3 antibody–based LDT has the potential to standardize PD-L1 scoring using the Ventana BenchMark XT or Benchmark ULTRA platforms, thereby serving an unmet need in pathology laboratories for PD-L1 testing.

Perhaps the best evidence of the equivalence of the two assays is the excellent correlation for CPS as a continuous variable (Figs 2 and 3). Although clinical interpretation of CPS ultimately leads to dichotomization of results around a cutoff to make a therapeutic decision, measures of concordance (NPA, PPA, and OPA) are influenced as much by the distribution of CPS values in proximity to the cutoff as by the analytic performances of the assays themselves. This agreement between two successive assays has been demonstrated by a mathematical model, which also showed that the clinical accuracy of dichotomized results can tolerate quite a bit of measurement uncertainty [17]. These findings are consistent with the notion that the LDT and PD-L1 IHC 22C3 pharmDx may be equally predictive of response to pembrolizumab, although they certainly do not offer proof. Of note, this is a limitation of most analytic concordance studies.

Published literature shows that a few other 22C3 antibody–based LDT protocols have been evaluated in NSCLC (using TPS) [18–20], gastroesophageal cancer (using CPS) [21], HNSCC (using TPS and CPS) [12], and TNBC (using TPS and CPS) [22]. Although high concordance between the LDT and PD-L1 IHC 22C3 pharmDx was reported for NSCLC and gastroesophageal cancer in the published reports [18–21], the concordance was found to be poor in HNSCC (ICC = 0.46 [TPS]; 0.34 [CPS]) [12] and variable in TNBC (ICC = 0.59 [TPS]; 0.48 [CPS]) [22], possibly because the LDT used the SP263 assay. The SP263 assay differs from the LDT we used by the primary antibody (SP236 vs 22C3) and the chemistry employed (OptiView vs ultraView plus amplification). In TNBC, higher concordance was established between the 28–8 and 22C3 antibody assays (ICC = 0.88 [TPS]; 0.84 [CPS]) [22]. To our knowledge, this is the first pan-tumor analytic comparison of an LDT and PD-L1 IHC 22C3 pharmDx across multiple tumor types.

Potential limitations of this study include the relatively small sample set sizes for CC (n = 77) and ESCC (n = 80) compared with those for TNBC (n = 118 [BenchMark XT]; n = 30 [BenchMark ULTRA]), UC (n = 121), and HNSCC (n = 126). However, the analytic comparability of the LDT used in this study was already successfully assessed in NSCLC (using TPS) and the analytic comparability in this study was successfully assessed in TNBC, UC, and HNSCC (using CPS). For this reason, we determined that an assessment of the smaller sample set sizes for CC and ESCC was sufficient because the goal was to ensure that the protocol did not fall outside of a reasonable confidence level. We did not report on the NPA for CC data because of the high prevalence of CPS ≥1 (positive) in the CC samples, which dictated that a much larger cohort would be needed for the NPA to be calculated. This study also did not consider interobserver variability, given that all samples were scored by a single experienced pathologist.

In conclusion, our 22C3 antibody–based LDT on the BenchMark XT or BenchMark ULTRA platforms demonstrates high concordance with PD-L1 IHC 22C3 pharmDx for the assessment of PD-L1 CPS across multiple tumor types, including CC, ESCC, HNSCC, TNBC, and UC. These findings suggest that the 22C3 antibody–based LDT on the BenchMark XT or BenchMark ULTRA platforms performs comparably with the gold-standard PD-L1 IHC 22C3 pharmDx for the assessment of PD-L1 status and can therefore be used to identify patients who are indicated for pembrolizumab therapy. Lastly, findings from our study will help laboratories who do not have access to the approved companion diagnostic assay for pembrolizumab (PD-L1 IHC 22C3 pharmDx and/or the DAKO Autostainer platform) to use the validated protocol for using 22C3 antibody on a Ventana BenchMark XT platform (and Ventana BenchMark ULTRA platform for TNBC) and enable them to use existing platforms without requiring the purchase of new equipment.

## References

[1] Pardoll DM. The blockade of immune checkpoints in cancer immunotherapy. Nat Rev Cancer. 2012; 12(4):252–64. doi: 10.1038/nrc3239 . 22437870 PMC4856023

[2] Iwai Y, Ishida M, Tanaka Y, Okazaki T, Honjo T, Minato N. Involvement of PD-L1 on tumor cells in the escape from host immune system and tumor immunotherapy by PD-L1 blockade. Proc Natl Acad Sci U S A. 2002; 99(19):12293–7. doi: 10.1073/pnas.192461099 . 12218188 PMC129438

[3] KEYTRUDA^®^(pembrolizumab) injection, for intravenous use. 4/2023. Merck Sharp & Dohme LLC, Rahway, NJ, USA; 2023.

[4] Keytruda (pembrolizumab) 50 mg powder for concentrate for solution for infusion (summary of product characteristics). 06/2022. MSD B.V., Haarlem, Netherlands; 2022.

[5] Emancipator K. Keytruda and PD-L1: a real-world example of co-development of a drug with a predictive biomarker. AAPS J. 2020; 23(1):5. doi: 10.1208/s12248-020-00525-1 . 33222057

[6] Ferris RL, Blumenschein G Jr., Fayette J, Guigay J, Colevas AD, Licitra L, et al. Nivolumab vs investigator’s choice in recurrent or metastatic squamous cell carcinoma of the head and neck: 2-year long-term survival update of CheckMate 141 with analyses by tumor PD-L1 expression. Oral Oncol. 2018; 81:45–51. doi: 10.1016/j.oraloncology.2018.04.008 . 29884413 PMC6563923

[7] Rui X, Gu TT, Pan HF, Zhang HZ. Evaluation of PD-L1 biomarker for immune checkpoint inhibitor (PD-1/PD-L1 inhibitors) treatments for urothelial carcinoma patients: a meta-analysis. Int Immunopharmacol. 2019; 67:378–85. doi: 10.1016/j.intimp.2018.12.018 . 30584967

[8] Agilent Technologies. PD-L1 IHC 22C3 pharmDx Rx Only SK006 50 tests for use with Autostainer Link 48. Agilent Technologies: Carpinteria, CA; 2020: 37.

[9] Neuman T, London M, Kania-Almog J, Litvin A, Zohar Y, Fridel L, et al. A harmonization study for the use of 22C3 PD-L1 immunohistochemical staining on Ventana’s platform. J Thor Oncol. 2016; 11(11):1863–8. doi: 10.1016/j.jtho.2016.08.146 27664534

[10] Kulangara K, Zhang N, Corigliano E, Guerrero L, Waldroup S, Jaiswal D, et al. Clinical utility of the combined positive score for programmed death ligand-1 expression and the approval of pembrolizumab for treatment of gastric cancer. Arch Pathol Lab Med. 2019; 143(3):330–7. doi: doi: 10.5858/arpa.2018-0043-OA . 30028179

[11] Nowicki TS, Hu-Lieskovan S, Ribas A. Mechanisms of resistance to PD-1 and PD-L1 blockade. Cancer J. 2018; 24(1):47–53. doi: 10.1097/PPO.0000000000000303 . 29360728 PMC5785093

[12] de Ruiter EJ, Mulder FJ, Koomen BM, Speel EJ, van den Hout M, de Roest RH, et al. Comparison of three PD-L1 immunohistochemical assays in head and neck squamous cell carcinoma (HNSCC). Mod Pathol. 2021; 34(6):1125–32. doi: 10.1038/s41379-020-0644-7 . 32759978

[13] Cogswell J, Inzunza HD, Wu Q, Feder JN, Mintier G, Novotny J, et al. An analytical comparison of Dako 28–8 PharmDx assay and an E1L3N laboratory-developed test in the immunohistochemical detection of programmed death-ligand 1. Mol Diagn Ther.. 2017; 21(1):85–93. doi: 10.1007/s40291-016-0237-9 . 27667773 PMC5250639

[14] Adam J, Le Stang N, Rouquette I, Cazes A, Badoual C, Pinot-Roussel H, et al. Multicenter harmonization study for PD-L1 IHC testing in non-small-cell lung cancer. Ann Oncol. 2018; 29(4):953–8. doi: 10.1093/annonc/mdy014 . 29351573

[15] Nambirajan A, Husain N, Shukla S, Kumar S, Jain D. Comparison of laboratory-developed test & validated assay of programmed death ligand-1 immunohistochemistry in non-small-cell lung carcinoma. Indian J Med Res. 2019; 150(4):376–84. doi: 10.4103/ijmr.IJMR_367_18 . 31823919 PMC6902360

[16] Torlakovic E, Lim HJ, Adam J, Barnes P, Bigras G, Chan AWH, et al. "Interchangeability" of PD-L1 immunohistochemistry assays: a meta-analysis of diagnostic accuracy. Mod Pathol. 2020; 33(1):4–17. doi: 10.1038/s41379-019-0327-4 . 31383961 PMC6927905

[17] Emancipator K. Does reproducibility drive clinical accuracy? Am J Clin Pathol. 2021; 156(4):577–85. doi: 10.1093/ajcp/aqaa267 . 33738478

[18] Ilie M, Khambata-Ford S, Copie-Bergman C, Huang L, Juco J, Hofman V, et al. Use of the 22C3 anti-PD-L1 antibody to determine PD-L1 expression in multiple automated immunohistochemistry platforms. PLoS One. 2017; 12(8):e0183023. doi: 10.1371/journal.pone.0183023 . 28797130 PMC5552229

[19] Ilie M, Juco J, Huang L, Hofman V, Khambata-Ford S, Hofman P. Use of the 22C3 anti-programmed death-ligand 1 antibody to determine programmed death-ligand 1 expression in cytology samples obtained from non-small cell lung cancer patients. Cancer Cytopathol. 2018; 126(4):264–74. doi: 10.1002/cncy.21977 . 29411536

[20] Hendry S, Byrne DJ, Wright GM, Young RJ, Sturrock S, Cooper WA, et al. Comparison of four PD-L1 immunohistochemical assays in lung cancer. J Thorac Oncol. 2018; 13(3):367–76. doi: 10.1016/j.jtho.2017.11.112 . 29175115

[21] Kim J, Kim B, Kim E, Jang M, Cho JH, Lee HS, et al. Interchangeability of PD-L1 laboratory-developed test by 22C3 antibody concentrate among ihc platforms in gastric cancer. Pathology. 2020; 52:S120. doi: 10.1016/j.pathol.2020.01.408

[22] Noske A, Ammann JU, Wagner DC, Denkert C, Lebeau A, Sinn P, et al. A multicentre analytical comparison study of inter-reader and inter-assay agreement of four programmed death-ligand 1 immunohistochemistry assays for scoring in triple-negative breast cancer. Histopathology. 2021; 78(4):567–77. doi: 10.1111/his.14254 . 32936950

